# Experimental and clinical evidences for glucose control in intensive care: is infused glucose the key point for study interpretation?

**DOI:** 10.1186/cc13998

**Published:** 2014-07-23

**Authors:** Aurélien Mazeraud, Andrea Polito, Djillali Annane

**Affiliations:** Institut Pasteur, Human Histopathology and Animal Models, Pr Chretien F, 28, rue du docteur Roux, 75015 Paris, France; Université Paris Descartes Sorbonne Paris Cité, Institut Pasteur, rue du Dr Roux, 75015 Paris, France; Medical and Surgical Intensive Care Unit, CHU Raymond Poincaré (AP-HP), University of Versailles Saint Quentin en Yvelines, 104 boulevard Raymond Poincaré, 92380 Garches, France; Université de Versailles Saint-Quentin en Yvellines, 55 Avenue de Paris, 78000 Versailles, France

## Abstract

Stress-induced hyperglycemia has been considered an adaptive mechanism to stress up to the first intensive insulin therapy trial, which showed a 34% reduction in relative risk of in-hospital mortality when normalizing blood glucose levels. Further trials had conflicting results and, at present, stress-induced hyperglycemia management remains non-consensual. These findings could be explained by discrepancies in trials, notably regarding the approach to treat hyperglycemia: high versus restrictive caloric intake. Stress-induced hyperglycemia is a frequent complication during intensive care unit stay and is associated with a higher mortality. It results from an imbalance between insulin and counter-regulatory hormones, increased neoglucogenesis, and the cytokine-induced insulin-resistant state of tissues. In this review, we summarize detrimental effects of hyperglycemia on organs in the critically ill (peripheric and central nervous, liver, immune system, kidney, and cardiovascular system). Finally, we show clinical and experimental evidence of potential benefits from glucose and insulin administration, notably on metabolism, immunity, and the cardiovascular system.

## Introduction

In an ICU, stress induces insulin resistance and overproduction of glucose, resulting in a syndrome called stress-induced hyperglycemia (SIH) [1]. SIH is common during critical illness and is associated with high mortality [1–3]. Its incidence is approximately 50% in septic shock [1] and 13% in surgical patients [4]. Up to the beginning of the 21st century, hyperglycemia was considered an adaptive mechanism to stress. In 2001, the landmark Leuven study by Van den Berghe and colleagues [4] reported a 34% relative risk reduction of in-hospital mortality when blood glucose was maintained at between 80 and 110 mg/dL. Since then, glucose metabolism during critical illness has been the focus of an increasing number of experimental and clinical studies. A decade later, after seven additional major randomized control trials, physicians remain confused about how to manage SIH. The heterogeneity of studies includes differences in population, ICU setting, staff experience, feeding strategy, blood glucose monitoring, variability, definition of hypoglycemia, insulin protocol, infusion site, its continuation after ICU discharge, and finally differences in the choice of the relevant major outcome. In the two ‘positive’ trials from Leuven, mean non-protein daily caloric intake was approximately 20 kcal/kg per day, essentially via glucose administration initially given intravenously: up to 200 to 300 g/day in the 2001 trial, with a median total daily insulin administration of 71 units (confidence interval of 48 to 100). By contrast, in NICE-SUGAR (Normoglycemia in Intensive Care Evaluation and Surviving Using Glucose Algorithm Regulation), which suggested increased mortality with intensive insulin therapy, caloric intake was 11.04 ± 6.08 kcal/kg per day, with 19.5% given intravenously, and cumulative mean daily dose of insulin was 50.2 ± 38.1 units per day [5]. Thus, there were two markedly different therapeutic approaches - that is, intensive gluco- and insulin therapy (liberal glucose intake, or the Leuven approach) and intensive insulin therapy (IIT) (restrictive glucose intake, or the NICE-SUGAR approach) [6]. The aim of this review is to discuss experimental evidence of organ injury and insulin sensitivity during SIH and expose differences in strategies for its control that include a liberal or a rather restrictive glucose intake.

## The risk associated with hyperglycemia

After several large clinical trials, there is still no consensus on what blood glucose level (BGL) is ‘too much’. Whereas the Leuven trial demonstrated deleterious effects from uncontrolled glucose levels, subsequent trials comparing strategies to control BGL reported conflicting results [4, 5, 7–11].

SIH is undoubtedly associated with mortality in stroke [12], brain injury [2], and myocardial infarction [13] patients; trauma, cardiothoracic surgery, thermally injured, and mixed ICU patients [3]; and non-ICU hospitalized patients. The aim of this section is to summarize current knowledge about the mechanisms of hyperglycemia toxicity.

### Pathogenesis of stress-induced hyperglycemia

Critical illness is characterized by an imbalance between insulin and endogenous or exogenous counter-regulatory hormones (glucagon and glucocorticoids). As a result, glucose production is increased and its storage is decreased secondary to downregulated glycogen synthesis and enhanced glycogenolysis [1]. In animal models, adrenaline infusion induces hyperglycemia via stimulation of hepatic and renal neoglucogenesis [14]. This pathway represents the major source of endogenous glucose during critical illness [1].

SIH is also the consequence of insulin resistance [1]. Experimental data on the mechanisms of sepsis-induced acute insulin resistance are scarce. Most of the knowledge about the mechanisms of acute insulin resistance comes from trauma/hemorrhage experimental models and studies in type 2 diabetes [15]. Under healthy conditions, insulin binding to its receptor results in the phosphorylation of insulin receptor substrates, which transmit insulin metabolic and growth signals. One major effector of the metabolic pathway is glucose transporter family (GLUT) 4, which facilitates glucose transport across cell membranes in muscle and adipose tissue. During injury, inhibitor of kappa B kinase and Jun B pathways are activated, leading to expression of inflammatory markers such as TNF. First, Jun B negatively regulates insulin receptor substrate and then TNF downregulates GLUT 4 gene transcription [16]. These mechanisms could account for insulin resistance during sepsis [17].

Whether this mechanism of response to aggression is deleterious during critical illness is still a matter of debate [18]. In fact, insulin resistance is variable from one tissue to another and appears to be moderate in the heart and diaphragm but is major in skeletal muscles and adipocytes [19]. In fact, despite this insulin resistance, glucose utilization is enhanced in sepsis secondary to different GLUT overexpression [20].

Glucose transport across cell membranes is the rate-limiting step of cellular glucose metabolism. Each GLUT is characterized mostly by organ specificity, insulin sensitivity, and the Michaelis constant (K_m_). K_m_ is defined as the transporter’s specific value of glycemia for which 50% of transport capacities are reached. These characteristics (Table 1) could explain differences in organ sensitivity to insulin and modulation of glucose uptake with glycemia. This heterogeneity may be seen as a protective effect of vital organs while placing other organs in a ‘hibernating state’.

**Table 1:** **Summary of different glucose transporter characteristics**

Transporter	Insulin sensitivity	Location	Michaelis constant (K_m_), mg/dL	Particularity
GLUT 4	Yes	Muscle, adipocytes	460	Accounts for insulin resistance. Glycemia-dependent transporter
GLUT 1	No	Blood–brain barrier, astrocytes, cardiomyocytes, liver, endothelium	25	Rate-limiting step of glucose transport in brain. Upregulated up to 1.7-fold in sepsis
GLUT 2	No	Liver, kidney, beta pancreatic cells	300	Glycemia-dependent transport
GLUT 3	No	Brain	25	Second important transporter in brain

GLUT, glucose transporter.

Hyperglycemia is responsible for reactive oxygen species (ROS) overproduction in diabetes via four major pathways: advanced glycated endproduct release, *de novo* indirect activation of C kinase protein, increased polyol, and hexosamine pathway flux redox homeostasis [21]. On one hand, some ROS production can be essential for immune cell ‘respiratory burst’ to kill pathogens or for endothelial cell functions. On the other hand, during hyperglycemia, excess ROS may worsen organ failure [22].

### Hyperglycemia and the central nervous system

Brain cells are dependent on glucose to maintain their membrane ionic gradient. However, the detrimental cerebral effects of hyperglycemia have been observed in critical illness [2, 12, 23].

Brain glucose metabolism has some particularities:

- Glucose crosses blood–brain barrier and cellular membranes via a high-affinity and insulin-insensitive process involving GLUT 1 and GLUT 3 [24].
- Neurons and astrocytes cooperate to metabolize carbohydrates, as suggested by lactate shuttles between these cells.
- During hypoxemic or hypoperfusion stress, GLUT 1 and 3 are upregulated (up to 300% in trauma) with a subsequent increase in glucose uptake [25].

Hyperglycemia has been shown to enhance the breakdown of the blood–brain barrier via induction of matrix metalloproteinase [26] and to induce apoptosis [23], mostly via enhanced superoxide production. Indeed, in epidemiologic studies on stroke, hyperglycemia is associated with edema, infarct size, mortality in non-diabetic patients, and poor functional status at 1 year [12]. Nevertheless, trials aiming at controlling BGL in stroke, subarachnoid hemorrhage [27], brain injury [28], and neuro-intensive care [29] patients did not report improved outcome with tight glucose control.

### Hyperglycemia and the peripheral nervous system

Neuromyopathy is a frequent complication of critical illness, such as septic shock and acute respiratory distress syndrome, with an incidence of up to 50% of patients with these conditions. In the first Leuven study, the risk of developing a critical illness neuromyopathy (CINM) was lowered from 49% to 25% (*P* < 0.0001) in the interventional group, facilitating weaning from mechanical ventilation. The mechanisms of hyperglycemic neuromyopathy are poorly understood and may involve activation of apoptotic and inflammation pathways in response to acute hyperglycemia in muscles [30] or ROS overproduction as suggested in type 2 diabetic neuropathy or CINM pathogenesis [21].

### Hyperglycemia and other organs

#### Liver

In resting conditions, GLUT 2 is the predominant transporter for glucose in hepatic parenchymal cells [24, 31]. This low-affinity transporter modulates glucose transport proportionally to BGL. After lipopolysaccharide (LPS) stimulation, GLUT 2 decreases, whereas GLUT 1 increases [31], resulting in an enhanced insulin- and glycemia-independent uptake of up to 2.4-fold [31, 32]. Analyses of liver cells from the control group of the Leuven study revealed dramatic lesions to the mitochondria. These mitochondria abnormalities may result from excessive glucose uptake, with subsequent overproduction of ROS [33], which could have been diminished with BGL normalization.

#### Immune system

In the Leuven study, patients with normoglycemia had almost 50% fewer bloodstream infections (7.8% versus 4.2%, *P* = 0.003) [4]. Indeed, each step of the immune response to stress is altered with hyperglycemia. First, in diabetes, chronic high BGL induces overexpression of surface and circulating cell adhesion molecules [34–36], whereas LPS challenge is less effective in upregulating cell adhesion molecules [34]. This enhanced overall immune cell adhesion paradoxically results in a less effective chemotactism and transmigration capacity of immune cells [37]. Second, worse polymorphonuclear killing capacities against pathogens, as assessed by concentrations of lysosomal enzyme or burst respiratory intensity, are observed when BGL is poorly controlled with a dose-effect relationship in diabetes [38]. Finally, the production of chemokines and other pro-inflammatory factors is decreased under hyperglycemic conditions [39].

#### Kidney

In septic shock, GLUT 2 and 3 expressions are decreased in the tubular epithelial cells of the kidney, whereas GLUT 1 expression is increased. This may account for enhanced glycosuria and acute renal failure during septic shock [40]. In the Leuven study, renal replacement therapy was twice less frequent in patients with normoglycemia, whereas insulin *per se* was associated with worse renal outcome [41]. This kidney protection may result directly from lower BGL, since high BGL directly inhibits transcription of an anti-apoptotic gene in renal tubules [42] or from improvement in lipid profile, ROS production, and endothelial protection [43].

#### Heart and endothelium

SIH has been shown to be an important prognostic factor in acute coronary syndromes [13]. The heart has a remarkable ability to switch from free fatty acid oxidation to carbohydrate oxidation under hypoxemic conditions [44]. During acute myocardial infarction, SIH activates T cells in the atherosclerotic plaque and increases tissue levels of inflammatory markers and nitric oxide and ROS production, resulting in endothelial dysfunction [45]. Consequently, coronary blood flow and reserve during myocardial infarction are impaired [46]. Furthermore, acute hyperglycemia increases infarct size and suppresses cardioprotective signal transduction via mitochondrial potassium ATP channel inhibition [47].

In shock, although BGLs are high, glucose represents only 12% of substrate oxidation by cardiomyocytes [48]. Therefore, one could argue that hyperglycemia without insulin infusion does not confer a metabolic benefit and presents rather deleterious consequences on an ischemic heart.

## Evidence for gluco-insulinotherapy

### Clinical trial calendar

See Table 2.

**Table 2:** **Trials’ calendar**

Year	Trial
2001	The first Leuven RCT (1,548 patients) reported a 34% relative risk reduction in hospital mortality with maintenance of BGL of between 80 and 110 mg/dL [4].
2003	Krinsley [49], in an observational study (1,826 patients), confirmed the survival benefit associated with protocolized IIT targeting a BGL of less than 140 mg/dL.
2006	The second Leuven RCT (1,200 patients) confirmed a 10% absolute reduction in hospital mortality for long-stay medical ICU patients with maintenance of BGL of between 80 and 110 mg/dL [11].
2008	De la Rosa *et al*. [9] RCT (504 patients) failed to show any survival benefit in mixed ICU patients targeting BGL between 80 and 110 mg/dL but with less effective control.
	The VISEP study, with the same glycemic goals (537 patients with septic shock), was terminated prematurely because of an unacceptably high incidence of hypoglycemia (17.0% versus 4.1%; *P* < 0.001) and no evidence for survival benefit at 90 days (39.7% versus 35.4%; *P* = 0.31) [50].
	Arabi *et al*. [8] RCT (523 patients) also failed to show survival benefit (adjusted hazard ratio 1.09, 95% confidence interval 0.70 to 1.72) and showed increased hypoglycemic rates (28.6% versus 3.1% of patients; *P* < 0.0001).
	SPRINT (BGL goal of 72 to 110 mg/dL) is an observational study with historic control. Nutritional and insulin protocols provided less variable and tighter glucose control (standard deviation of blood glucose was 38% lower compared with the retrospective control) with subsequent improvement in organ failures and outcome for long-stay ICU patients: failure-free days were different (SPRINT = 41.6%; Pre-SPRINT = 36.5%; *P* < 0.0001) [51].
2009	The Glucontrol (1,101 patients) was stopped prematurely for unintended protocol violations. The IIT was associated with increased hypoglycemia (8.7% versus 2.7%; *P* = 0.0001) and a non-significant trend to higher mortality (15.3% versus 17.2%) while BGL was not optimally controlled [10].
2009	The NICE-SUGAR trial (6,104 mixed ICU patients) compared a strategy of BGL control of between 81 and 108 mg/dL versus a more liberal strategy (<180 mg/dL). This RCT found an increase in mortality with IIT (27.5 versus 24.9; *P* = 0.02) and increased incidence of hypoglycemia (6.8% versus 0.5%; *P* < 0.001) [7].
2010	COITTSS (509 patients with septic shock) compared a strategy of BGL control of between 80 and 110 mg/dL versus maintenance of BGL of less than 150 mg/dL. This trial did not find any difference in in-hospital mortality between the two strategies (45.9% versus 42.9%; *P* = 0.05) [8].

BGL, blood glucose level; COITTSS, Corticosteroids and Intensive Insulin Therapy for Septic Shock; IIT, intensive insulin therapy; NICE-SUGAR, Normoglycemia in Intensive Care Evaluation and Surviving Using Glucose Algorithm Regulation; RCT, randomized controlled trial; SPRINT, Specialized Relative Insulin and Nutrition Tables; VISEP, Efficacy of Volume Substitution and Insulin Therapy in Severe Sepsis.

### Analysis of critical differences between trials

In the Leuven studies, patients were given intravenous glucose at 8 to 12 g/hour, with mean intravenous glucose feeding of 120 g during the first 15 hours, to a goal of 200 to 260 g/day afterward. This study shows higher intravenous glucose and insulin administration than in any other study (Table 3), whereas epidemiologic studies have shown that both are correlated with an increased mortality [3, 41, 52].

**Table 3:** **Summary of characteristics from the different major trials about glucose-insulin treatment**

Study name	VDB 2001 [4]		VDB 2006 [11]		Glucontrol [10]		NICE-SUGAR [5]		COITTSS [7]		VISEP [50]	
	Experimental	Control	Experimental	Control	Experimental	Control	Experimental	Control	Experimental	Control	Experimental	Control
Morning mean BGL, mg/dL	103.6	154.5	110.0	160.9	110.9	140.0	118.0	144.9	147.3	154.5	112.7	152.7
SD or confidence interval	20.0	32.7	30.0	28.0	100-123.6	121.8- 160	25.1	26.0	30.9	34.5	1.8	3.6
Death at 90 days, %	5	7	35.9	37.7	23.3	19.4	27.5	24.9	45.9	42.9	39.7	35.4
Caloric intake, kcal/day	550-1,600		1,202	1,237	760	760	891	872	1,350		1,217	1,253
Quantity of glucose administered per day, g	120		202	198	73.7	71.8	23.4	24.4	25		144	144
Daily insulin dose, insulin units	71	33	59	10	31.2	7.68	50.2	16.9	71	46	43	29
SD or confidence interval	48-100	17-56	37-86	0-38	15.6- 55.2	30.48	38.1	29	45-96	30-65	23-64	15-51
Hypoglycemia rate, %	0.8	5	18.7	3.1	8.7	2.7	6.8	0.5	16.4	7.8	10.1	4.1

BGL, blood glucose level; COITTSS, Corticosteroids and Intensive Insulin Therapy for Septic Shock; NICE-SUGAR, Normoglycemia in Intensive Care Evaluation and Surviving Using Glucose Algorithm Regulation; SD, standard deviation; VDB, Van den Berghe; VISEP, Efficacy of Volume Substitution and Insulin Therapy in Severe Sepsis.

Other trials of glucose control in the ICU used lower glucose intake and insulin doses. In none of these studies did the experimental intervention achieve maintenance of normal BGL like in the Leuven study. NICE-SUGAR is the only study showing increased mortality with tight BGL control. In that study, total caloric intake was much lower than in the Leuven study. In the Specialized Relative Insulin and Nutrition Tables (SPRINT) study, a 35% lowering of hospital mortality for patients with a long stay in the ICU (*P* = 0.02) was observed after implementation of tight glucose control when glucose was administered enterally to allow a caloric intake of 25 kcal/kg per day. Likewise, the cumulative insulin dose per day was close to that observed in the experimental group of the Leuven study (67.2 units in SPRINT versus 71 units). These findings are in line with the latest IIT meta-analysis by Marik and Preiser [6], who suggested that intravenous calorie administration plays a pivotal role for improvement of outcome during IIT. In contrast, the last Leuven trial, EPaNIC (Early versus late Parenteral Nutrition in Intensive Care), showed that parenteral nutrition administration to achieve a caloric intake of 20 to 25 kcal/kg per day might be detrimental. This raises the question of the effect of an exclusive and important glucose infusion during IIT in critical illness.

It was then suggested that, in the Leuven trial, difference in observed mortality was secondary to a higher mortality in the control group due to an excessive glucose load. Nevertheless, control mortality in the Leuven study matched the mortality expected from estimation of the EuroSCORE (European System for Cardiac Operative Risk Evaluation). Secondly, a recent meta-analysis suggested that intravenous glucose intake was an independent predictive factor for good outcome in the Leuven studies [6]. But whether blood glucose control or insulin administration mediated positive effects in this study was not studied.

In 2003, Van den Berghe and colleagues [41] performed a *post-hoc* analysis of their first study. The authors showed that both total amount of infused insulin and glycemic control were associated with lower mortality (independently of age, delayed ICU admission, Acute Physiology and Chronic Health Evaluation II score, reason for ICU admission, history of malignancy or diabetes, and at-admission hyperglycemia). The strength of association between the mortality rate and the mean BGL seemed to be stronger than with the total daily infused insulin [41]. Nevertheless, no statistical comparison was made between these factors in this study. Furthermore, the respective effects of these two entwined factors could be analyzed only in an interventional study comparing gluco-insulinotherapy versus tight glycemic control. Then a recent study by Arabi and colleagues [53] with a 2 × 2 factorial design compared IIT and permissive underfeeding (60% to 70% of daily recommended caloric intake versus 90% to 100%). Their study showed no mortality differences between groups but was underpowered and non-blinded, and the therapeutic goals were not achieved [53].

Finally, in 2011, the Leuven group performed the EPaNIC study that evaluated the timing of parenteral nutrition introduction. In that study, a strategy of early parenteral nutrition initiation was performed with administration of 400 kcal (100 g) at day 1 and 800 kcal at day 2 exclusively via intravenous glucose administration, and then a relay with mixed parenteral and enteral nutrition was performed to achieve calculated daily physiological caloric intake [54]. The control group received minimal glucose administration and enteral nutrition was started at day 2 if oral intake was insufficient. Results showed an increased rate of complications in the parenteral nutrition group (infection and cholestasis), whereas the late initiation of parenteral nutrition resulted in a shorter duration of renal replacement therapy, mechanical ventilation, and stay in the ICU. In that study, the amount of administered glucose was three times lower than in the 2001 study, and insulin doses were also lower in both groups: 31 insulin units (interquartile range (IQR) 19 to 48) in the control versus 58 insulin units (IQR 40 to 85) in the experimental group. Furthermore, parenteral nutrition contains lipid at recommended doses that could present detrimental effects as fat oxidation is a high oxygen-consuming metabolic pathway. A *post-hoc* analysis of EPaNIC concerning the first 2 days in the ICU in that study before introduction of parenteral nutrition might be of interest to clinicians and help them determine whether high glucose administration during IIT is beneficial for patients. We will present clinical and experimental evidence that may support the use of a glucose-insulin administration strategy.

### Is gluco-insulinotherapy associated with a decreased incidence of hypoglycemia?

The clinical signs of hypoglycemia are commonly masked in sedated patients. Thus, in clinical trials, hypoglycemia was defined empirically by a BGL value of less than 40 mg/dL. Its incidence varied from 5.1% to 18.7% in patients with IIT and from 0.5% to 4.1% in control groups. Seizures and comas have occasionally been observed following severe hypoglycemic episodes without establishing a clear causal relationship [55]. Neuronal death during or following hypoglycemia has also been found in both animal and human models, but hypoglycemia does not seem to affect neurocognitive development in children [56] but may contribute to long-term cognitive impairment following critical illness in adults [57]. The existence of a direct causal link between hypoglycemia and mortality remains controversial, and hypoglycemia could reflect only a more severe illness. Some epidemiologic studies have found that only early or spontaneous hypoglycemia was independently associated with death in critically ill patients [58, 59]. Preventive interventions are thus warranted in such a situation. Whether an increased daily amount of carbohydrate administered would decrease the risk of hypoglycermia during tight BGL is unknown.

In a retrospective study by Arabi and colleagues [60], glucose intake was not a risk or protective factor of hypoglycemia whereas insulin daily dosage was an evident risk factor (73.5 ± 36.7 in the group presenting hypoglycemia versus 47.5 ± 51.8; *P* < 0.0001) [60]. Actually, caloric intake lowering (gastroparesis, intravenous glucose, or enteral nutrition lowering) without insulin adjustment may be one of the most frequent risk factors for hypoglycemia [54, 55, 60, 61], and no study evaluated the effect of gluco-insulinotherapy on hypoglycemia rate. The recent EPaNIC study showed a decreased rate of hypoglycemia during IIT when early parenteral nutrition was initiated (1.9% versus 3.5%, *P* = 0.001), suggesting a possible protective role of gluco-insulinotherapy to be explored in an interventional study [54].

### Effects of high insulin and glucose intake on organs

Gluco-insulinotherapy consists of a high amount of glucose infused and higher insulinemia. Effects of insulin on glycemia lowering are mediated mostly by an increase in cellular uptake of glucose through GLUT 4 translocation to the membrane. GLUT 4 is located mostly on adipocytes and skeletal muscle cells [1]. Thus, mostly GLUT 4-expressing cells consume glucose administered intravenously during IIT.

During early sepsis, metabolic stress resulted in glycogenolysis and depleted energetic reserves as shown in skeletal muscle biopsies [33, 62]. This ATP depletion was correlated with poor outcome, and in addition recovery from sepsis was preceded by normalization of the phosphocreatine/ATP ratio [62]. Indeed, energy depletion during sepsis could be a risk factor for CINM, an ICU complication associated with higher mortality [63]. In fact, skeletal muscle protein levels were higher in the IIT group consistently with anabolic effects of insulin [33]. Insulin augments the number of GLUT 4 receptors in adipose and skeletal muscle cells and the glucose uptake by myocytes [64]. This higher amount of energetic substrate may be a protective factor against energy depletion and sarcopenia and may lower the risk to develop CINM. These findings are in line with the observed lower rate of CINM and the late improvement in survival in the Leuven trials [4, 11].

Experiments from the Leuven cohort showed that maintenance of normal BGL protected the liver and skeletal muscles. In the liver, glucose uptake through GLUT 2 is independent of BGLs. Indeed, untreated hyperglycemia was associated with severely damaged mitochondria with altered complex I and IV activities [33]. Furthermore, insulin administration during injury partly suppresses gluconeogenesis [65]. Gluconeogenesis is an active process occurring mostly in the liver that requires four molecules of ATP and two molecules of GTP. This energy requirement may enhance hypoxic injury in liver during stress and could be counteracted with insulin administration [64–66].

Whether gluco-insulinotherapy rather than BGL control protects the liver from hypoxic injury has been studied in few human and experimental studies. One study conducted by a different group showed beneficial effects of insulin independently of BGLs on hepatocyte apoptosis, cytolysis, and expression of inflammatory markers [65]. Further studies in the Leuven group did not reproduce these results and showed a lower blood level of transaminases in burn-injured rabbits with BGL control rather than insulin administration [67]. Liver injury may be mediated by a mitochondriopathy in reaction to cellular hyperglycemia and enhanced glycolysis and is likely to mediate organ damage [68]. As insulin sensitivity is not overcome during IIT in the liver, glucose uptake by hepatocytes is likely to be dependent on glycemia rather than insulinemia [66].

In sepsis models, insulin has been shown to improve immune cell function independently of glycemia. It inhibits the apoptosis of activated macrophages, may modulate antigen presentation, and improves chemotaxis and phagocytic properties. Finally, insulin may modulate the balance between lymphocyte T helper type and lymphocyte T helper type 2 cells, favoring anti-inflammation and repair function [69]. Such effects of insulin on the immune system may account for the reduced rate of bloodstream infection during IIT [4].

During sepsis, the heart shows little or no insulin resistance [70] and lowers its glucose consumption [48, 70]. In porcine models, glucose and insulin infusion favored glucose and lactate utilization. This results in improvement in inotropic function without higher oxygen consumption observed in different studies [67, 71]. In fact, during acute coronary syndrome, glucose insulin potassium therapy was associated with substantial survival and may prevent arrhythmias [72]. However, the benefit of glucose insulin potassium infusion in patients with myocardial infarction remains controversial. In the ICU, IIT was not associated with reduced time or doses of inotropic support [4] and was associated with a higher incidence of cardiovascular death in NICE-SUGAR [5], but the Leuven study introduced IIT with an important amount of intravenous glucose administered to cardiovascular post-surgical patients. Such an inotropic effect may have improved organ perfusion and contributed to the lower renal failure rate and the better outcome in these patients.

In summary, gluco-insulinotherapy may present protective effects on muscle or improve immune or cardiac function. Contrary to Marik and Bellomo [18] in their recent comment, we hypothesize that gluco-insulinotherapy may be a more beneficial rather than a restrictive strategy. This issue needs to be further studied.

## Conclusions

The era of glucose control in the ICU started in 2001. Untreated SIH no doubt favors morbidity and mortality. Critical analyses of randomized controlled trials have suggested that glucose control is more likely to be associated with survival benefit when strict normal glucose levels are achieved and early high glucose intake is provided. An interventional study evaluating liberal and restrictive glucose intake during IIT is warranted to provide reliable evidence.

## Abbreviations

BGL: blood glucose level
CINM: critical illness neuromyopathy
EPaNIC: Early versus late Parenteral Nutrition in Intensive Care
GLUT: glucose transporter
IIT: intensive insulin therapy
LPS: lipopolysaccharide
NICE-SUGAR: Normoglycemia in Intensive Care Evaluation and Surviving Using Glucose Algorithm Regulation
ROS: reactive oxygen species
SIH: stress-induced hyperglycemia
SPRINT: Specialized Relative Insulin and Nutrition Tables
TNF: tumor necrosis factor.
